# Anti-Hypochlorite, Antioxidant, and Catalytic Activity of Three Polyphenol-Rich Super-Foods Investigated with the Use of Coumarin-Based Sensors

**DOI:** 10.3390/biom10050723

**Published:** 2020-05-06

**Authors:** Karolina Starzak, Tomasz Świergosz, Arkadiusz Matwijczuk, Bernadette Creaven, Janusz Podleśny, Dariusz Karcz

**Affiliations:** 1 Department of Analytical Chemistry (C1), Faculty of Chemical Engineering and Technology, Cracow University of Technology, Warszawska 24, 31-155 Cracow, Poland; tomasz.swiergosz@pk.edu.pl; 2 Department of Biophysics, University of Life Sciences in Lublin, Akademicka 13, 20-950 Lublin, Poland; arkadiusz.matwijczuk@up.lublin.pl; 3 School of Chemical and Pharmaceutical Sciences, Technological University Dublin, Kevin St., Dublin 2, Ireland; bernie.creaven@tudublin.ie; 4 Institute of Soil Science and Plant Cultivation—State Research Institute, 24-100 Puławy, Poland; jp@iung.pulawy.pl

**Keywords:** anti-hypochlorite activity, fluorescent probes, coumarin sensors, ABTS, green synthesis, açaí, goji, schisandra, super-foods, antioxidants

## Abstract

The anti-hypochlorite activity of açaí (*Euterpe oleracea* Mart.), goji (*Lycium barbarum* L.) and schisandra (*Schisandra chinensis*) fruit extracts were assessed by determining the reactive chlorine species (RCS)-scavenging ability of these three “super-food” berries. In addition, the aqueous extracts obtained were employed as both the media and the catalyst in a green chemistry approach to the synthesis of a coumarin-based fluorescence turn-off sensor, which was then used for anti-hypochlorite activity testing. The aqueous extracts were also assessed for total phenolic content (TPC), using the Folin–Ciocalteu method, and the antioxidant activity using the ABTS^+•^ assay. Moreover, the main water-soluble polyphenolic constituents of the extracts were identified by the HPLC-PDA-ESI-MS technique. Among the extracts tested, açaí demonstrated the highest anti-hypochlorite and antioxidant activities, while the highest TPC value was found for the goji extract. All extracts demonstrated modest catalytic activity as Knoevenagel condensation catalysts.

## 1. Introduction

Hypochlorous acid (HOCl) is synthetized in vivo as a result of an enzymatic reaction catalyzed by myeloperoxidase within activated neutrophils [1]. HOCl plays a critical role in the microbial killing and acts in a nonspecific, first-stage human immune response [2], and thus is important for the protection against a wide range of pathogens. However, its nonspecific response means that it acts as a mediator of tissue damage and results in inflammation, causing damage to cellular components such as nucleic acids, proteins, cellular organelles, or tissues, surrounding places of its origin in vivo [2]. Recent reports have identified that chlorinative stress may lead to the development of neurodegenerative diseases [3]. In particular, the accumulation of HOCl becomes even more problematic in the case of people diagnosed with chronic inflammatory conditions, whose immune system is constantly stimulated towards increased HOCl production, while the physiological hypochlorite-scavenging system, based mostly on glutathione (GSH) [3,4] is not able to effectively eliminate its excesses.

The anti-hypochlorite activities of various plant extracts are well known [1,4,5] and are also of interest to our group. For instance, our previous studies on betalains-rich red beetroot (*Beta vulgaris* L.) extract, demonstrated the ability to scavenge hypochlorite [6,7], and was shown to reduce knee discomfort associated with osteoarthritis [8,9]. Other reports suggest that polyphenol-rich natural products can assist in the elimination of HOCl in vitro [5,10,11,12].

These experiments are in line with our more recent research interest, namely biologically active coumarin derivatives and especially those which may serve as fluorescent sensors specific for selected molecules/radicals. Recently, the interest in fluorescent markers and probes, which are highly specific to hypochlorous acid, has increased with numerous contributions from the field of coumarin chemistry [13,14,15]. A representative example of such coumarin derivatives is 7-diethylamino-coumarin-3-carboxylic acid (7-DCCA) [16,17], well-known for its interesting, excited state properties, which critically include that its fluorescence quantum yield is highly dependent on its environment [18]. The reports on properties and potential practical applications for 7-DCCA are consistent with our own results, where the fluorescence-quenching-based mechanism of hypochlorite sensing by 7-DCCA was proposed, rendering this compound an efficient turn-off fluorescence probe for hypochlorite ions in vitro [13]. Moreover, this probe was successfully applied by our group for an assessment of the anti-hypochlorite activity of a *Moringa oleifiera* food supplement [19].

Upon interaction with hypochlorous acid, the fluorescence emission of 7-DCCA is quenched, and the quenching rate depends linearly on the concentration of hypochlorous acid. Although the proposed mode of action involved an electrophilic substitution occurring at the C4 position of 7-DCCA with the formation of 4-chloro-7-diethylaminocoumarin [13], our work identified the formation of 8-chloro-substituted 7-DCCA as the main product. Importantly, the chlorinated derivatives formed are non-fluorescent upon excitation at the wavelength used to excite the highly fluorescent 7-DCCA [13]. The nature of this phenomenon is not fully explained; however, considering the relative bulkiness of the chlorine substituent, it is likely that the lack of fluorescence results from spin-orbit coupling, which favors the relaxation via intersystem crossing to the excited triplet state [20,21].

The results obtained to date prompted us to extend our investigation into the applicability of the 7-DCCA as a hypochlorite sensor in the assessment of the pro-health properties of the selected super-foods, namely the açaí (*Euterpe oleracea* Mart.), goji (*Lycium barbarum* L.), and schisandra (*Schisandra chinensis*). Due to their high polyphenol content, these berries demonstrate a number of beneficial protective effects on human tissue acting via an antioxidative or an anti-inflammatory mode of action [22,23,24,25,26]. The high antioxidant capacity of açaí, goji, and schisandra berries is a characteristic and well-known property of these fruits. In recent years, a large number of publications focusing on the biological and pro-healthy activities of these berries has been published. However, in most of the cases, the high activity observed is due to the fact that pure organic solvents or their mixtures were used for the extraction of active compounds from plant material [27,28,29,30,31,32,33,34].

Our studies were focused on the physiological conditions occurring in the human body, and since 70–85% of the human body consists of water [35], we decided to conduct the measurements under conditions that were as close as possible to the physiological conditions. Also, the extraction using organic solvents would not be an accurate representation of the concentration of polyphenols, which would be available through normal ingestion. Therefore, all berries were extracted with water, and the extracts obtained were subjected to antioxidative activity testing. It is also worth mentioning that the use of alcoholic extracts for the scavenging of reactive chlorine species such as hypochlorite would result in an undesirable reaction between the alcohol and hypochlorous acid. This, in turn, would result in the formation of chlorinated derivatives such as chloroform, hydrochloric acid chloroacetone, dichloroacetate, and many other toxic species. Therefore, the use of solvents other than water, and especially alcohol in the determination of anti-hypochlorite activity was not considered.

The main aim of our current study was to determine the anti-hypochlorite activity of berries mentioned. Their extraordinary pro-health properties categorize these fruits as super-foods and result in their growing popularity, which during the last few years resulted in their global use as food additives. In order to study their activity in conditions that best mimic the physiological environment, all experiments were performed using aqueous extracts. The total phenolic content (TPC) using the Folin–Ciocalteu method and the antioxidant activity of the berries extracts using the ABTS^+•^ assay were determined whilst the main water-soluble polyphenolic constituents of the extracts were identified with the use of HPLC-PDA-ESI-MS technique. Complementarily, a green synthesis approach was applied for the isolation of 7-DCCA as a product of the Knoevenagel condensation between 4-diethylamino-2-hydroxybenzaldehyde and Meldrum’s acid.

## 2. Materials and Methods

### 2.1. Materials

All solvents were of 99% purity or higher (HPLC grade and MS grade). All chemicals used were of a reagent grade or higher. ABTS (2,2′-azino-bis(3-ethylbenzothiazoline-6-sulphonic acid)), Folin–Ciocalteu reagent, TROLOX (6-hydroxy-2,5,7,8-tetramethylchroman-2-carboxylic acid), gallic acid, 4-diethylamino-2-hydroxybenzaldehyde, Meldrum’s acid were purchased from Sigma Aldrich (St. Louis, MO, USA). Salicylic acid, chlorogenic acid, caffeic acid, rutin, *p*-coumaroylquinic acid, quercetin-3-*O*-galactoside, vanillic acid, orientin, and phloretin were purchased from Merck (Darmstadt, Germany). Sodium hypochlorite was purchased from ChemPur (Piekary Śląskie, Poland). Methanol, ethanol, formic acid, acetonitrile, and sodium carbonate were purchased from Avantor (Gliwice, Poland). Acetonitrile hypergrade and formic acid for LC-MS analysis were supplied by Merck (Darmstadt, Germany). The demineralized water used throughout the experiments was distilled through a Purix water purification system (Purix, Copenhagen, Denmark).

The plant material was imported from local markets in Brazil (açaí) and China (goji and schisandra). Goji were purchased as whole, freeze-dried fruits, while the acai and schisandra were purchased as freeze-dried powders. The samples were delivered to the laboratory in a frozen state and were kept that way until analysis.

### 2.2. Instrumentation

UV-Vis spectrophotometric and steady-state fluorescence measurements were performed on a Tecan Infinite 200 microplate reader (Tecan Austria GmbH, Grödig/Salzburg, Austria).

The FT-IR spectra were recorded in the region of 4000 cm^−1^ to 450 cm^−1^ on a Shimadzu IR Spirit Fourier-Transform Infrared spectrophotometer equipped with an ATR adapter (Shimadzu, Kyoto, Japan).

Mass spectrometry analysis was performed on Shimadzu 8030 ESI-Triple Quad mass spectrometer (Shimadzu, Kyoto, Japan).

### 2.3. Methods

#### 2.3.1. Extracts Preparations for Total Phenolic Content, Antioxidant and Anti-Hypochlorite Analysis

The efforts at optimizing the experimental procedure in terms of mimicking the physiological conditions of human body were made by preparing the aqueous extracts without the addition of any organic solvents. On the other hand, in order to eliminate the undesired side-effects associated with the presence of various interfering species, demineralized water was used for the extraction. In the case of açaí and Schisandra, 100 mg of freeze-dried powders were suspended in 10 mL of demineralized water. In the case of goji berries, 1 g of freeze-dried, whole fruits were put into a mortar and ground with a pestle until a homogeneous powder was obtained. Then 100 mg of ground sample was suspended in 10 mL of demineralized water. All three suspensions were then vigorously shaken and kept for extraction at room temperature for 15 min, and then centrifuged at 5000 rpm for 10 min. The supernatants were collected and stored at −18 °C for total phenolic content determination, antioxidant, and anti-hypochlorite assays.

#### 2.3.2. Total Phenolic Content

The aqueous extracts were examined for their total phenolic content (TPC) with the use of a modified Folin–Ciocalteu assay. The Folin–Ciocalteu reagent (0.5 mL) was mixed with a fixed volume of extract (2.5 mL) and was kept at 25 °C for 3 min, followed by the addition of saturated sodium carbonate (1.5 mL). Samples were incubated at 40 °C for 30 min, and then their absorbance was measured at λ_max_ 765 nm. The results were expressed as gallic acid equivalents (GAE) per 1 g of the sample dry weight.

#### 2.3.3. Antioxidant Assay

Increasing concentrations of each extract (10 mg/mL) were applied to transparent 96-well plates so that their final volume at each pH separately, was set to decrease the radicals absorbance in the range of 10–90% of its initial intensity. Samples were buffered with 20 μL of 25 mM acetate (pH 3 and 5) or phosphate (pH 7.4) buffers. Samples of the reference compound, which was TROLOX (TROL), were prepared the same way, and their final concentration ranged from 0 to 17.5 mg/mL in 200 μL of the total volume of each sample. Prior to the measurement, all wells were supplemented with 40 μL of 1 mM aquatic solution of ABTS^+•^ radicals. The total volume of each sample was 200 μL. To ensure thorough mixing of reagents in all wells, each plate was shaken on an internal shaker of the reader for 10 s. The spectrophotometric measurements were performed in the range of λ 350–750 nm with a 1 nm wavelength step at 25 °C for 30 min. The results obtained were the average of five exposures of each sample with a beam of light. All experiments were replicated three times.

#### 2.3.4. Green Synthesis of 7-DCCA Fluorescent Probe

Synthesis of 7-DCCA probe was carried out according to the previously reported procedure with minor modifications [19]. The powdered plant material (1 g) was suspended in demineralized water (100 mL) and sonicated for 10 min. The solution was then filtered off and used as the medium for the isolation of the 7-DCCA probe.

Meldrum’s acid (0.75 g, 5.0 mmol) was added to the suspension of 4-diethylamino-2-hydroxybenzaldehyde (1.0 g, 5 mmol) in the aqueous extract. The mixture was stirred overnight at 20 °C, and the orange solid was then filtered off and recrystallized from methanol, resulting in the formation of crystalline 7-DCCA. The identity and purity of the product isolated were confirmed by HPLC-MS and referenced to our previously obtained data [13,19].

#### 2.3.5. Anti-Hypochlorite Assay

To the wells of a black, 96-well plate, 20 μL of 25 mM acetate (pH 3 and 5) or phosphate (pH 7.4) buffers, 80 μL of extracts tested (10 mg/mL), and increasing concentrations of NaOCl were injected. Hypochlorite concentration in the total volume (200 μL) of samples ranged from 0 to 360 μM. Mixtures were incubated for 15 min at room temperature, and the next 30 μL of 1 mM 7-DCCA ethanolic probe solution was added to each sample. Each plate was shaken for 10 s on the reader shaker, and then the fluorescence measurements started. All samples were excited by light at λ_Exc_ 289 nm [13,19], and the fluorescence emission was recorded within the range of λ 320–700 nm at 25 °C. The results obtained were the average of five exposures of each sample with a beam of light. All experiments were repeated three times. Analysis of the blank samples, without the addition of any extract, and for the reference substance TROLOX, 80 μL (0.25 mg/mL) of the methanolic solution in each well was made analogously.

#### 2.3.6. Preparation of Standard Solutions and Plant Extracts for LC-MS/MS Analysis

A solution of salicylic acid, chlorogenic acid, caffeic acid, rutin, *p*-coumaroylquinic acid, quercetin-3-*O*-galactoside, vanillic acid, orientin, and phloretin was prepared in demineralized water by weighing out 5 mg of the analyte into 50 mL volumetric flask. All standard solutions were stored in the dark at 5 °C. A solution of the aqueous extract of açaí, goji, schisandra at the concentration of 500 mg/mL was sonicated in an ultrasonic bath for 30 min. The samples were stored in the dark at low temperature (5 °C) for additional identification of individual polyphenols. Before HPLC-DAD-ESI-MS analysis, all solutions (mixed standards and samples were filtered through a 0.20 µm nylon syringe filter (Sartorius, Germany) and then degassed in an ultrasonic bath for 15 min.

#### 2.3.7. LC-MS/MS Conditions for Polyphenols Determination

The phenolic content of the aqueous açaí, goji, and schisandra extracts were determined by HPLC-DAD-ESI-MS. All experiments were performed on an LCMS-8030 mass spectrometric system (Shimadzu, Kyoto, Japan) coupled to an LC-20ADXR Nexera pump (Shimadzu, Kyoto, Japan) with an injector model SIL-20ACXR (Shimadzu, Kyoto, Japan) and electrospray ionization (ESI). The LC-MS system was controlled with the LabSolutions software version 5.91 SP1 (Shimadzu, Japan). Samples were eluted through a 100 mm × 4.6 mm i.d., 5.0 μm Kinetex C18 chromatographic column (Phenomenex, Torrance, CA, USA) proceeded by a 4 mm × 2 mm i.d. guard column of the same material (Phenomenex, Torrance, CA, USA). The column was thermostated at 35 °C. All samples before analysis were diluted in demineralized water and centrifuged at 3000× *g* for 10 min. The injection volume was 50 μL, and the flow rate was 0.5 mL/min for all the analyses. The analysis was performed with a binary gradient system. The mobile phases were A—0.1% (*v/v*) formic acid in water, and B—acetonitrile. The gradient profile was: (*t* [min], % B), (0, 0), (2, 0), (13, 3), (28, 12), (35, 23), (44, 24), (52, 38), (66, 90). The ionization electrospray source operated in negative mode (ESI^−^), at an electrospray voltage of 5 kV and capillary temperature at 250 °C, using N_2_ as a gas for the spray (15 L/min), recording total ion chromatograms, mass spectra, and the selected ion monitoring (SIM). The mass spectrometer was operated in the scan mode from *m/z* 10 to 2000.

## 3. Results and Discussion

### 3.1. Total Phenolic Content in Aqueous Extracts of the Açaí, Goji, and Schisandra Berries

The total phenolic content (TPC) of the berries tested here has been previously assessed by other groups and has a very broad spectrum of values [27,28,29,36,37]. The differences are mainly due to the solvent or solvent mixtures used for the extraction of polyphenols from plant material. In this context, the treatment of the plant material prior to the extraction process should also be taken into account. For example, in the case of açaí berries, the TPC values vary from 312 mg GAE (gallic acid equivalents) per gram in lyophilized, oil-free açaí powder, purified from water-soluble compounds via solid phase extraction (SPE) prior to TPC determination [36], to that of 13.9 mg GAE/g in freeze-dried-only berries [27]. In both cases, organic solvents were used.

**Figure 1:**
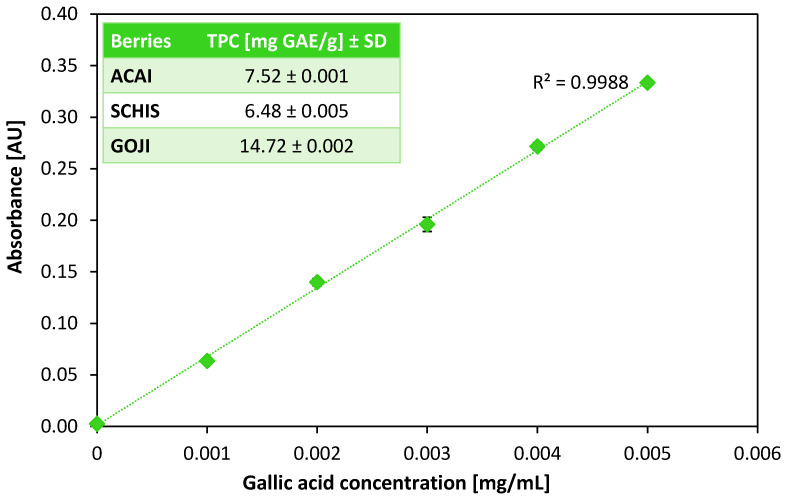
Calibration curve for gallic acid (GA). Absorbance was measured at λ 765 nm at 25 °C. Inset: values of total phenolic content in aqueous extracts from three tested berries species. (Data are expressed as mean ± standard deviation (n = 3)).

Since all three berries species tested do not require any treatment before consumption, in our studies, the aqueous extracts were used without any additional preparatory steps. The total phenolic content was determined using the colorimetric, Folin–Ciocalteu assay. Gallic acid (GA) was used as a reference compound. The calibration curve was prepared in a range of 0–0.005 mg GA/mL (R^2^ = 0.9988). The TPC in 1 g of each extract was calculated and is presented as an inset in Figure 1.

The highest polyphenol concentration, 14.72 mg GAE/g was obtained for the goji berries. This may be a result of the fact that whole berries (ground prior to the extraction) were taken, whilst in the case of the açaí and schisandra plants, the material bought was already in powdered form. It is very likely that the outer skin of the fruit can protect natural compounds present in the interior from environmental factors such as atmospheric oxygen or solar radiation, hence the higher polyphenol content in the case of goji berries. Islam et al. determined phenolic content in two types of goji berries—red (*Lycium barbarum*) and black (*Lycium ruthenicum*), and the TPC values they obtained were in the range of 2.1–3.1 mg GAE/g and 7.2–9.0 mg GAE/g for red and black berries, respectively [28]. However, the authors used a mixture of acetone/water/acetic acid for extraction, which makes the comparison with the results obtained from an aqueous extract difficult. Our own results suggest that in an aquatic environment, goji berries are able to release a high number of polyphenols.

Schauss et al. also used a mixture of acetone/water/acetic acid as an extracting solution for the determination of TPC in açaí (*Euterpe oleracea*) [27]. Ferreira et al. used a mixture of ethanol and 1.5 M HCl to extract polyphenols from different fractions of açaí berries [37]. The respective results obtained (13.9 mg GAE/g and 13.8 mg GAE/g) are slightly higher compared to those obtained in our study (7.52 mg GAE/g) and are likely a result of the more efficient extraction of polyphenols with the use of a mixture of acidified organic solvents compared to that of water.

Wang [29], prior to TPC determination, extracted *Schisandra chinensis* at 80 °C in pure ethanol, and the extract obtained was extracted again using a series of solvents with increasing polarity (from petroleum ether to water). The highest polyphenol content, determined in the ethyl acetate fraction, was 102.54 mg GAE/g, while in the aqueous fraction, polyphenols were not determined. A total of 2.74 mg GAE/g of polyphenols were determined in fruits of *Schisandra chinensis* by Mocan [38] after methanol was used for the extraction from the berries. Those values are comparable with those obtained in our study, 6.48 mg GAE/g.

All the above results were obtained after extraction of the plant material with water and are similar to those from organic solvents, indicating that the aqueous physiological conditions are capable of quite effective polyphenol extraction. Moreover, the TPC values obtained correspond well with the IR-spectroscopic data (see Section 3.5).

### 3.2. Antioxidant Assay

The antioxidant capacity was measured according to the methodology proposed by Miller [39] using ABTS^+•^ radicals and with TROLOX (TROL) as an accepted standard. ABTS^+•^ is a water-soluble, stable radical cation obtained in the reaction of ABTS with sodium persulfate. During the reaction with potential antioxidants, the blue-green color of the radical solution turns colorless, which can be monitored spectrophotometrically at λ_max_ 734 nm. The rate of the decolorization reaction indicates the strength of the potential tested antioxidant.

**Figure 2:**
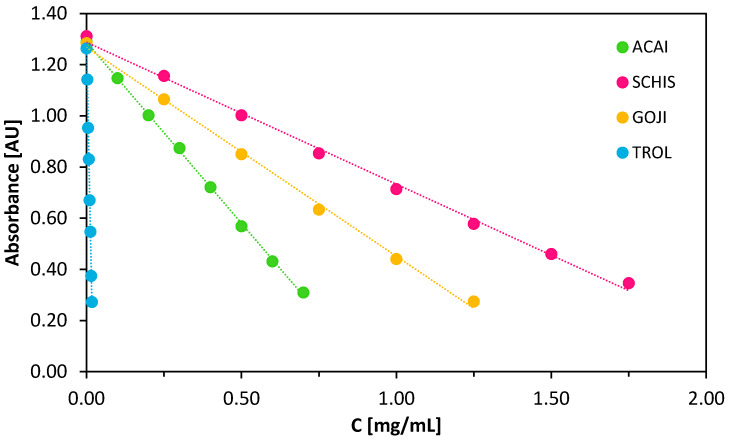
The decrease in ABTS^+•^ absorbance measured at λ_max_ 734 nm in the presence of increasing concentrations of the selected berry extracts and TROLOX after 30 min of reaction at pH 7.4 at 25 °C.

Figure 2 shows the decrease of absorption of ABTS^+•^ radicals’ solution at λ_max_ 734 nm after 30 min of incubation with increasing concentrations of the aqueous solutions of berries tested at pH 7.4.

Significant differences between the slopes of extracts and that of TROLOX indicate differences in the antioxidative activity of all samples tested. A significant decrease in absorption of ABTS^+•^ solution in the presence of low TROLOX concentration indicates its much higher ability to reduce radicals compared to that of other samples. The results obtained for pH 3 and 5 are presented in the Supplementary Material as Figure S1 and Figure S2, respectively.

**Figure 3:**
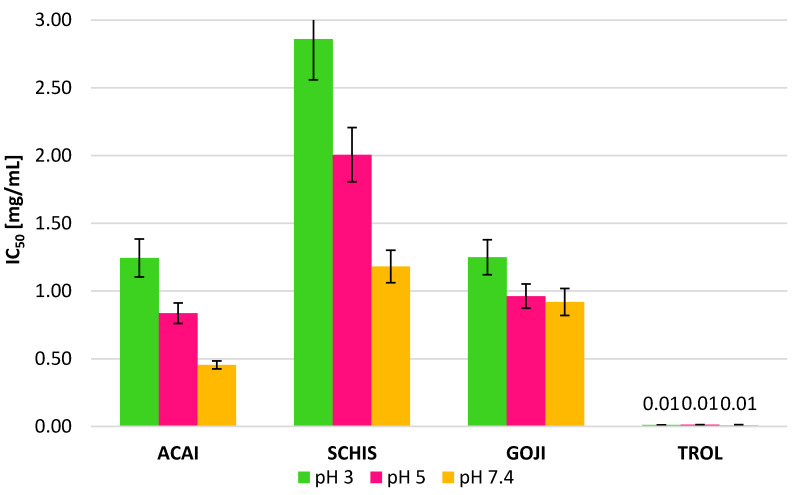
IC_50_ values of the extracts tested and the TROLOX at various pH values.

IC_50_ values were calculated in order to express and compare the radical scavenging potency of all the samples tested at various pH’s (Figure 3). The values obtained represent the concentration of the individual sample required to reduce 200 μM of ABTS^+•^ radicals by 50% in 30 min at room temperature.

In the case of TROLOX, the concentrations needed were in the range 0.011–0.013 mg/mL, while for the berry extracts, the IC_50_ values are in the range of 0.46 mg/mL for açaí at pH 7.4 to 2.86 mg/mL for Schisandra at pH 3. Recent studies, showing the effect of various solvents used for the extraction, on the ability of *Schisandra chinensis* berries extracts to scavenge 110 μM of DPPH^•^ (2,2-diphenyl-1-picrylhydrazyl) radicals, show the lowest antioxidant activity (IC_50_ 0.0703 mg/mL) of aqueous extracts [29]. These findings are in line with our results, where 1.18 mg/mL of the Schisandra extract at pH 7.4 demonstrated the ability to remove 50% of 200 μM of ABTS^+•^ radicals (Figure 3). The açaí berries showed the highest activity among species tested, probably as a result of the differences in the composition and the highest content of compounds with potential antioxidant activity.

Moreover, it is worth noticing that all the extracts tested exhibit an increase in their activity upon an increase in pH (lower IC_50_ values), which undoubtedly is a promising trend, especially once considered in the context of pH conditions prevailing in the human body.

### 3.3. Synthesis of Hypochlorite-Sensitive Fluorescent Probe 7-DCCA

**Figure 4:**
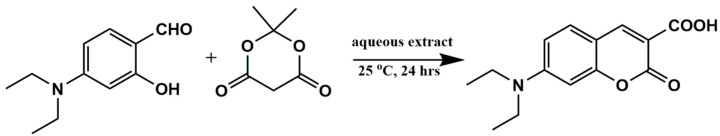
Aqueous berries extract-mediated synthesis of the fluorescent probe 7-DCCA.

The aqueous extracts were used as a catalyst and medium in the synthesis of the coumarin-derived fluorescent probe 7-DCCA (Figure 4). These experiments were inspired by the fact that a wide variety of crop-derived products, such as fruit juices or extracts, were employed as both media and catalysts in the synthesis of coumarin derivatives via the Knoevenagel condensation mechanism [40,41].

The use of açaí and schisandra extracts resulted in an identical yield of 7-DCCA obtained (28%), while in the case of the goji extract, it was slightly higher (31%). The reaction yields were referenced to that carried out in 10% of aqueous acetic acid and compared with our previously reported data [19]. All three extracts demonstrated significantly lower catalytic properties compared to that of the dilute acetic acid (56%). Moreover, the yields of 7-DCCA obtained with the use of the extracts tested are notably lower compared to that obtained as a result of our previous study [19]. Nevertheless, it is worth emphasizing that in all the cases, the unreacted salicylaldehyde substrate was recovered at the recrystallization step, making the attempted syntheses relatively cost-effective. On the other hand, no unreacted Meldrum’s acid was found, suggesting the possibility of side reactions involving the components of the extracts tested. These aspects, however, would require a more in-depth investigation and were not considered in our current work.

### 3.4. Anti-Hypochlorite Assay

The aqueous açaí, goji, and schisandra extracts were treated with increasing concentrations of sodium hypochlorite at pH 3, 5, and 7.4. After 15 min of the samples’ incubation at room temperature, a fluorescent probe (7-DCCA) sensitive to hypochlorite was added. Samples without the addition of the hypochlorous and extracts were used as the respective negative and positive control. The chosen pH values, in which the reactions were carried out, were dictated by the variation in pH occurring in the human body. For instance, pH 3 corresponds to conditions at the beginning of the digestive tract; the interior of the phagosomes, in which hypochlorite is formed in vivo, is an environment with a pH close to 5 [42,43] and a pH of 7.4 corresponds with most body fluids conditions.

**Figure 5:**
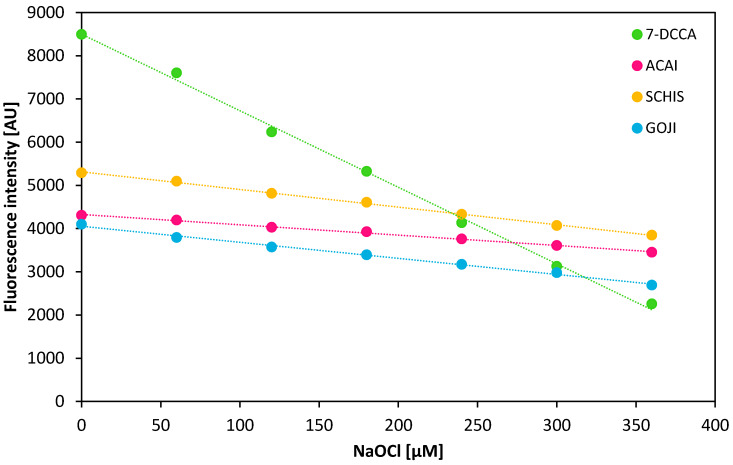
The decrease in fluorescence intensity of 150 μM of the 7-DCCA probe (λ_Ex_ 289 nm, λ_Em_ 460 nm) in the presence of the aqueous berry extracts (4 mg/mL) incubated with increased concentrations of NaOCl for 15 min at pH 7.4.

Changes in the fluorescence emission intensity at pH 7.4 in all extracts are presented in Figure 5. The results from measurements carried out at pH 3 and 5 are attached as Supplementary Material (Figure S3 and Figure S4), respectively.

The notable slowdown of the probes fluorescence intensity drop most likely resulted from the scavenging of the OCl^−^ ions by compounds present in the extracts. Considering the fact that the extracts were prepared in water to imitate (to some extent) the aqueous physiological conditions, the hypothesis is that water-soluble compounds are responsible here for such a significant lowering of the fluorescence quench speed rate. This, in turn, allows the assumption that the use of extracts prepared in less polar solvents, such as methanol, ethanol, or acetonitrile, would give even more satisfactory results. The investigation into organic solvent extracts was not the aim of this work. The most effective scavenging of OCl^−^ is observed in the açaí berries extract (the smallest slope angle), but the results obtained from goji and schisandra extracts are in the same range. The results suggest that in the human body, the water-soluble components of extracts absorbed into body fluids from the gastrointestinal tract would probably be able to eliminate hypochlorite, supporting our inborn physiological mechanisms of OCl^−^ removal. Secondly, the coumarin-derived sensors demonstrated the ability to penetrate cell membranes [44,45]. Thus, 7-DCCA can be applied as an intracellular marker for the presence and removal of hypochlorous acid. Yap et al. stated that up to 400 μM of HOCl is produced by activated neutrophils in every hour [3]. Table 1 presents the percentage of fluorescence drop by each extract after 15 min of incubation with 360 μM of NaOCl at all the pH’s tested.

The most significant difference between the reference sample and the extract samples is observed at pH 3, regardless of the fact that chances for the hypochlorite to react with the extract components under these conditions in vivo are very low. The most probable place for the contact of natural compounds occurring in extracts with hypochlorite is body fluids (pH 7.4). In aqueous solutions, HOCl may dissociate in a number of different ways depending on the pH of the environment. At pH 7.4, HOCl is almost in equilibrium with the highly reactive OCl^−^ ions [42]. Under these conditions, the açaí extract was the most effective. After only 15 min at room temperature, the fluorescence intensity drop observed was at approximately 20% of its initial value, for a high concentration of hypochlorite as 360 μM. In the reference sample (without extract added), the identical conditions allowed for ~73.4% of the probes fluorescence drop. In general, the berries’ extracts tested demonstrated the highest anti-hypochlorite activity at acidic conditions (4.0% of fluorescence drop at pH 3 for açaí) and the lowest at conditions similar to those of the physiological conditions (fluorescence drop to 34.3% of the initial value at pH 7.4 in goji extract) (Table 1). It is highly likely that the composition of extracts contributes to their overall anti-hypochlorite activity. The results obtained in the anti-hypochlorite tests coincide with those obtained from the antioxidative testing, and in both cases, the highest activity was observed in açaí extract. This points to the fact that açaí berries contain the highest amount of compounds capable of reducing and scavenging radicals that are potentially harmful to the human body.

### 3.5. IR Spectroscopy of Plant Powder Samples

**Figure 6:**
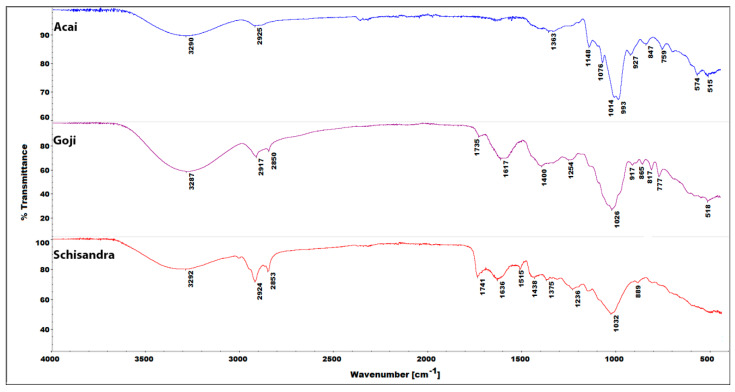
FT-IR (ATR) spectra of plant powder samples: açaí (top), goji (middle), schisandra (bottom).

The plant material used for the preparation of extracts was investigated by FT-IR spectroscopy (Figure 6). Previously, such studies carried out by our group proved useful in the qualitative analysis of honey [46], confirming its applicability in the analysis of foods and other materials of plant origin. Each spectrum shows moderate intensity and a broad band in the region of approximately 3300 cm^−1^, characteristic of intramolecular interactions via the -OH groups [47]. Additionally, this region is occupied by a series of somewhat sharper bands at ~2900 cm^−1^, which are most likely attributed to the C–H stretches, although the aliphatic chains of alcohols can also absorb in this region [47]. In the fingerprint region of the goji and schisandra powders, the spectra were dominated by intensive and sharp bands at 1030 cm^−1^, while in the case of açaí, this band was shifted towards lower wavenumbers by approximately 15 cm^−1^. In each case, these prominent bands may be assigned to the C–O stretching vibrations in aliphatic or alicyclic secondary alcohols, as well as various sugar residues. The phenolic C–O stretching vibrations are usually positioned at higher wavenumbers (~1300–1400 cm^−1^) [47]. Only the goji powder gave a distinct yet broad and moderate-intensity band at 1400 cm^−1^, while in the remaining powders, this region of the spectra is occupied by a series of moderate intensity bands suggesting the wide variety of polyphenolic species present. These results correspond well with those obtained from the TPC assay, where the highest number of polyphenols was determined in the goji extract, while the respective TPC contents in the açaí and schisandra extracts were notably lower (see Section 3.1). Interestingly, the spectra of the schisandra and goji powder revealed a relatively sharp band at 1741 and 1735 cm^−1^, respectively, suggesting the relatively high content of carbonyl moieties, and especially esters or carboxylic acids [47]. The band representing the carboxyl C=O stretching is most intensive in the schisandra extract, while in the goji extract, it is slightly less intense. The açaí spectrum did not show the band in this region, suggesting the lack of carboxylic acid and ester components.

### 3.6. Polyphenols Determination

The determination of polyphenolic constituents present in the berries tested has been widely reported together with the variety of solvents used for the extraction [22,30,33,38,48,49,50,51]. As previously mentioned, the main focus of our current study was an investigation into the aqueous berries’ extracts. Therefore, as expected, the comparison of our results to those previously reported revealed the presence of a relatively high amount of water-soluble compounds, while the absence of those with sparing aqueous solubility. It is particularly noteworthy that the relatively high content of phloretin was found in goji and schisandra extract, which to the very best of our knowledge, were analyzed for the first time for the presence of this compound. Phloretin (3-(4-hydroxyphenyl)-1-(2,4,6-trihydroxyphenyl)propan-1-one), commonly found in an apricot and apple leaves [52,53,54], has a very strong antioxidant potential. Additionally, it is involved in skin-brightening and participates in various DNA-repair processes occurring in skin cells, which suffered from sunlight overexposure. Also, phloretin shows effects very similar to those caused by ferulic acid. Therefore, the combined presence of phloretin, and ferulic acid may demonstrate the photoprotective effect, stronger than that of vitamin C [55,56]. Our results clearly indicate the presence of phloretin in (*m/z* 609) in schisandra and goji extracts (Figure S5), while its absence in açaí. Based on the existing reports [57,58], the presence of ferulic acid in the extracts tested was expected, although in our study, its content remained undetermined due to the low aqueous solubility of this compound. Several other natural compounds were found in all three aqueous berries’ extracts tested, namely the chlorogenic acid, salicylic acid, 2,4-dihydroxybenzoic acid, rutin (Figures S6–S9), *p*-coumaroylquinic acid, quercetin-3-*O*-galactoside. Moreover, 3-feruloylquinic acid was found in açaí and schisandra berries and vanillic acid and orientin in açaí. Their identification was based on the respective external standards run at conditions identical to those applied for the analysis of extracts (data not shown).

## 4. Conclusions

In conclusion, three aqueous extracts from popular, edible berries namely açaí, goji, and schisandra were tested for their anti-hypochlorite potency exhibiting the ability to effectively scavenge the hypochlorite ions in vitro. Only subtle differences were noticed between the anti-hypochlorite activities of the extracts; however, the açaí extract (4 mg/mL) proved most effective in removing OCl^−^ ions from samples after only 15 min of incubation with 360 μM hypochlorite at room temperature.

The ABTS^+•^ assay-based antioxidative activity testing of the extracts confirmed their ability to act as antioxidants with only slight differences observed between the activity values. Similar to the anti-hypochlorite activity, also, in this case, the açaí extract demonstrated the highest activity.

The comparison of TPC values of the extracts revealed a nearly two-fold higher content of polyphenols in goji berries, compared to those of açaí and schisandra. The TPC values determined corresponded well with the FT-IR spectra of the powdered berries. The value determined in the goji extract was 14.72 mg GAE/g, while in the açaí and Schisandra, these values were 7.52 and 6.48 mg GAE/g, respectively. Regardless of the subtle differences, the results obtained clearly point at the beneficial properties of the extracts and confirm the categorization of the examined berries as “super-foods”.

The catalytic activities of extracts were assessed by applying them as both media and catalysts in the synthesis of the highly fluorescent coumarin derivative 7-DCCA, which was then used during the anti-hypochlorite activity testing as a marker of the presence of OCl^−^ ions. In terms of the reaction conditions required for the synthesis of structurally different HOCl-sensitive fluorescent probes [59,60], a relatively low yield of the isolated 7-DCCA probe is not a drawback. The high purity of the 7-DCCA together with easy recovery of the unreacted substrate make the attempted syntheses economically advantageous. Overall, the results obtained with the use of 7-DCCA gave other evidence for the applicability of this compound as a hypochlorite-sensitive turn-off fluorescent probe.

As complementary work, the main phenolic constituents present in the aqueous extracts were identified using the HPLC-ESI-MS technique. The results obtained allowed for the quantification of a number of water-soluble polyphenols, such as chlorogenic acid, rutin, and 2,4-dihydroxy-benzoic acid. Moreover, presence of phloretin was confirmed for the first time. This is particularly worth-emphasizing given the fact that to date, this compound was confirmed to occur only in apple leaves and apricot [52,53,54].

## Figures and Tables

**Table 1:** The percentage drop in fluorescence intensity for all tested samples (4 mg/mL) under the influence of 360 μM of NaOCl after 15 min of incubation at 25 °C.

	Decrease of Fluorescence Intensity [%]		
Sample	pH 3	pH 5	pH 7.4
7-DCCA	92.1	72.0	73.4
açaí	4.0	22.8	19.9
goji	12.2	17.0	34.3
schisandra	21.7	20.3	27.2

## Supplementary Materials

The following are available online at https://www.mdpi.com/2218-273X/10/5/723/s1. Figure S1: Decrease of ABTS^+•^ absorbance measured at λ_max_ 734 nm in the presence of an increasing concentration of selected reagents after 30 min of reaction at pH 3 at 25 °C. Figure S2: Decrease of ABTS^+•^ absorbance measured at λ_max_ 734 nm in the presence of an increasing concentration of selected reagents after 30 min of reaction at pH 5 at 25 °C. Figure S3: Decrease of fluorescence intensity of 150 μM of 7-DCCA probe (λ_Ex_ 289 nm, λ_Em_ 460 nm) under the presence of aqueous berries extracts (4 mg/mL) incubated with an increasing concentration of NaOCl for 15 min at pH 3. Figure S4: Decrease of fluorescence intensity of 150 μM of 7-DCCA probe (λ_Ex_ 289 nm, λ_Em_ 460 nm) under the presence of aqueous berries extracts (4 mg/mL) incubated with an increasing concentration of NaOCl for 15 min at pH 5. Figure S5: LC-MS (SIM) chromatogram recorded in negative mode of aqueous extracts of goji and schisandra berries compared with a standard solution of phloretin. Figure S6: LC-MS (SIM) chromatogram recorded in negative mode of aqueous extracts of açaí, goji, and schisandra berries compared with a standard solution of chlorogenic acid. Figure S7: LC-MS (SIM) chromatogram recorded in negative mode of aqueous extracts of açaí, goji, and schisandra berries compared with a standard solution of salicylic acid. Figure S8: LC-MS (SIM) chromatogram recorded in negative mode of aqueous extracts of açaí, goji, and schisandra berries compared with standard solution of 2,4-dihydroxybenzoic acid. Figure S9: LC-MS (SIM) chromatogram recorded in negative mode of aqueous extracts of açaí, goji, and schisandra berries compared with a standard solution of rutin.
