# Supplementary material for: Anti-Hypochlorite, Antioxidant, and Catalytic Activity of Three Polyphenol-Rich Super-Foods Investigated with the Use of Coumarin-Based Sensors
Source: Biomolecules. 2020 May 6;10(5):723. doi: 10.3390/biom10050723 (PMC7277801; doi:10.3390/biom10050723)
Supplement: Supplementary file 1 [file biomolecules-10-00723-s001.pdf]

Supplementary materials for:

Anti-hypochlorite, antioxidant, and catalytic activity of three polyphenol-rich *super-foods* investigated with use of coumarin-based sensor

Karolina Starzak [1*], Tomasz Świergosz[1], Arkadiusz Matwijczuk[2], Bernadette Creaven[3], Janusz Podleśny[4] and Dariusz Karcz[1*]

1 Department of Analytical Chemistry (C1), Faculty of Chemical Engineering and Technology, Cracow University of Technology, Warszawska 24, 31-155 Cracow, Poland; karolina.starzak@pk.edu.pl, tomasz.swiergosz@pk.edu.pl, dariusz.karcz@pk.edu.pl

2 Department of Biophysics, University of Life Sciences in Lublin, Akademicka 13, 20-950 Lublin, Poland; arkadiusz.matwijczuk@up.lublin.pl

3 School of Chemical and Pharmaceutical Sciences, Technological University Dublin, Kevin St., Dublin 2, Ireland; Bernie.Creaven@tudublin.ie

4 Institute of Soil Science and Plant Cultivation - State Research Institute, Puławy, Poland; jp@iung.pulawy.pl

* Correspondence: karolina.starzak@pk.edu.pl (K.S); dariusz.karcz@pk.edu.pl (D.K);

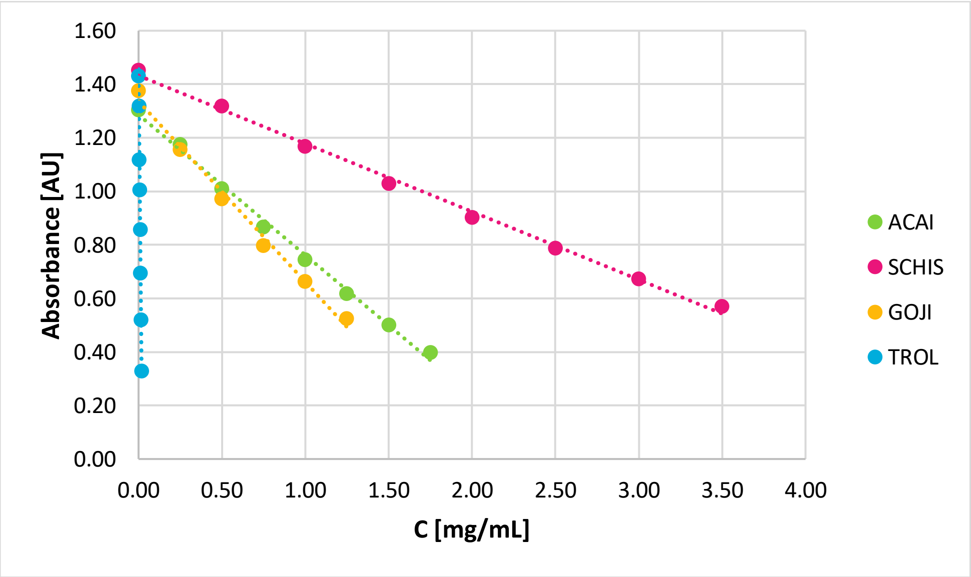

Figure S1. Decrease of $ABTS^{+\bullet}$ absorbance measured at λ_{max} 734 nm in the presence of increasing concentration of selected reagents after 30 min of reaction at pH 3 at 25 °C.

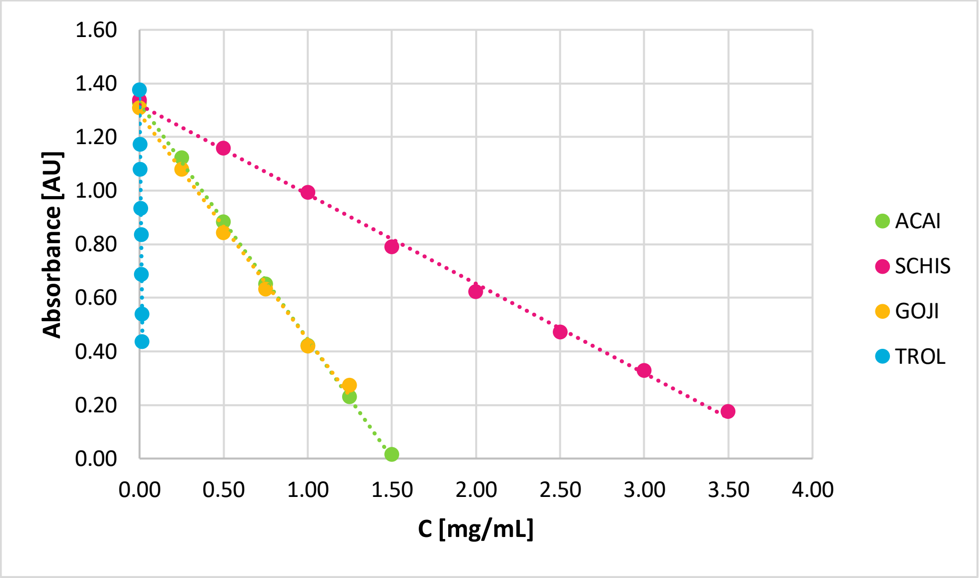

Figure S2. Decrease of ABTS$^{+\bullet}$ absorbance measured at λ_{max} 734 nm in the presence of increasing concentration of selected reagents after 30 min of reaction at pH 5 at 25 ^{o}C.

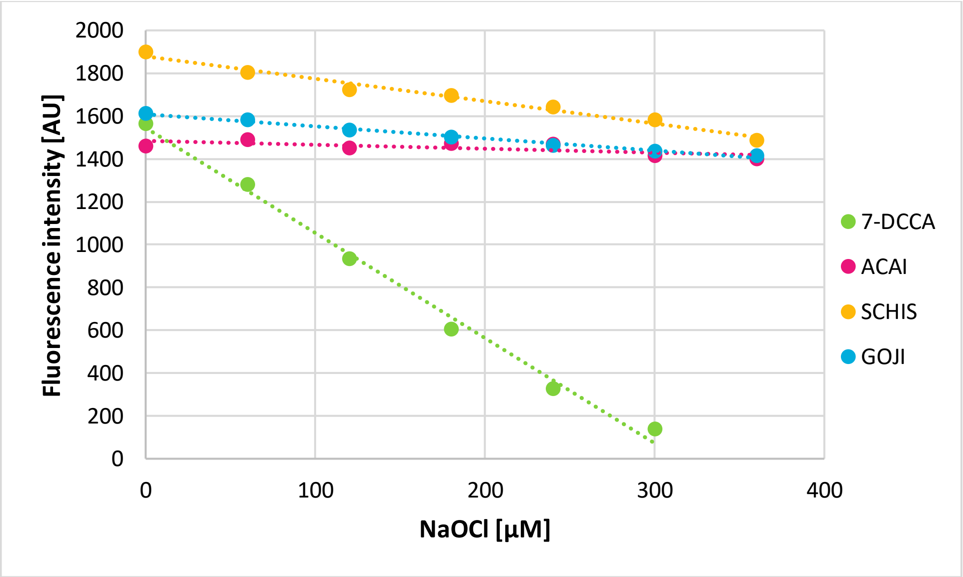

Figure S3. Decrease of fluorescence intensity of 150 μM of 7-DCCA probe (λ_{Ex} 289 nm, λ_{Em} 460 nm) under the presence of aqueous berries extracts (4 mg/mL) incubated with increasing concentration of NaOCl for 15 min at pH 3.

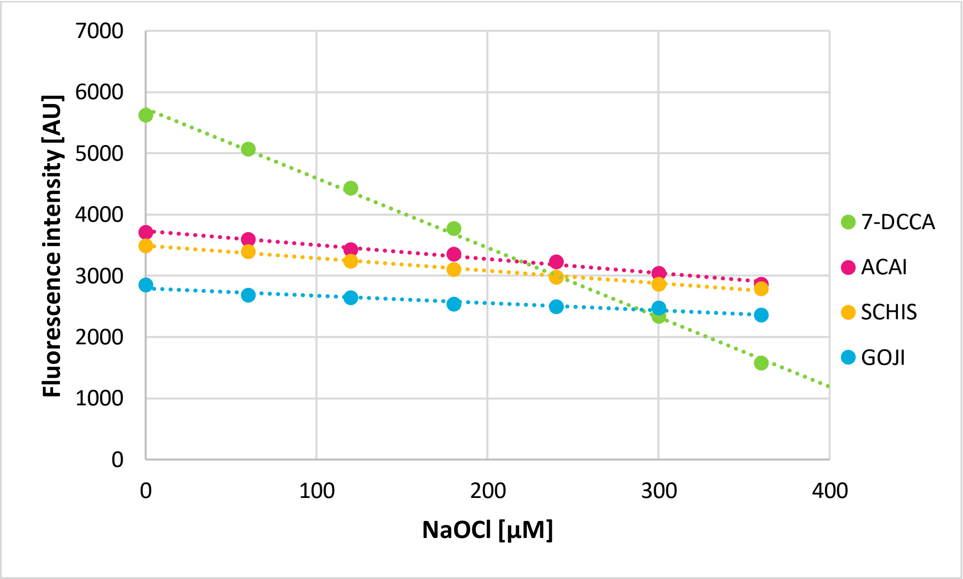

Figure S4. Decrease of fluorescence intensity of 150 µM of 7-DCCA probe (λ_{Ex} 289 nm, λ_{Em} 460 nm) under the presence of aqueous berries extracts (4 mg/mL) incubated with increasing concentration of NaOCl for 15 min at pH 5.

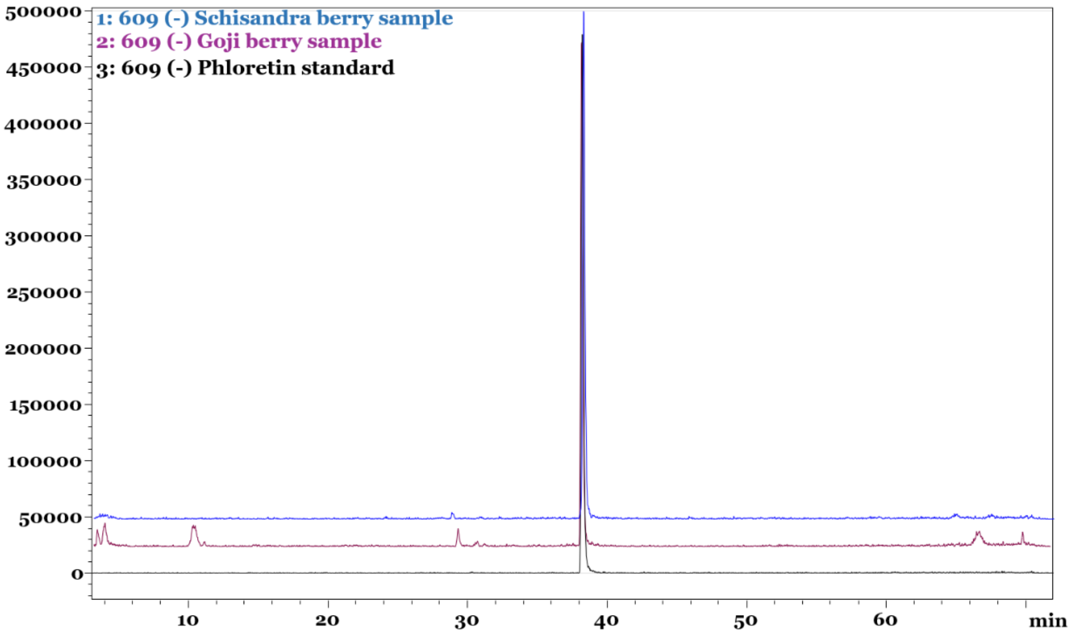

Figure S5. LC-MS (SIM) chromatogram recorded in negative mode, of an aqueous extracts of goji and schisandra berries compared with standard solution of phloretin.

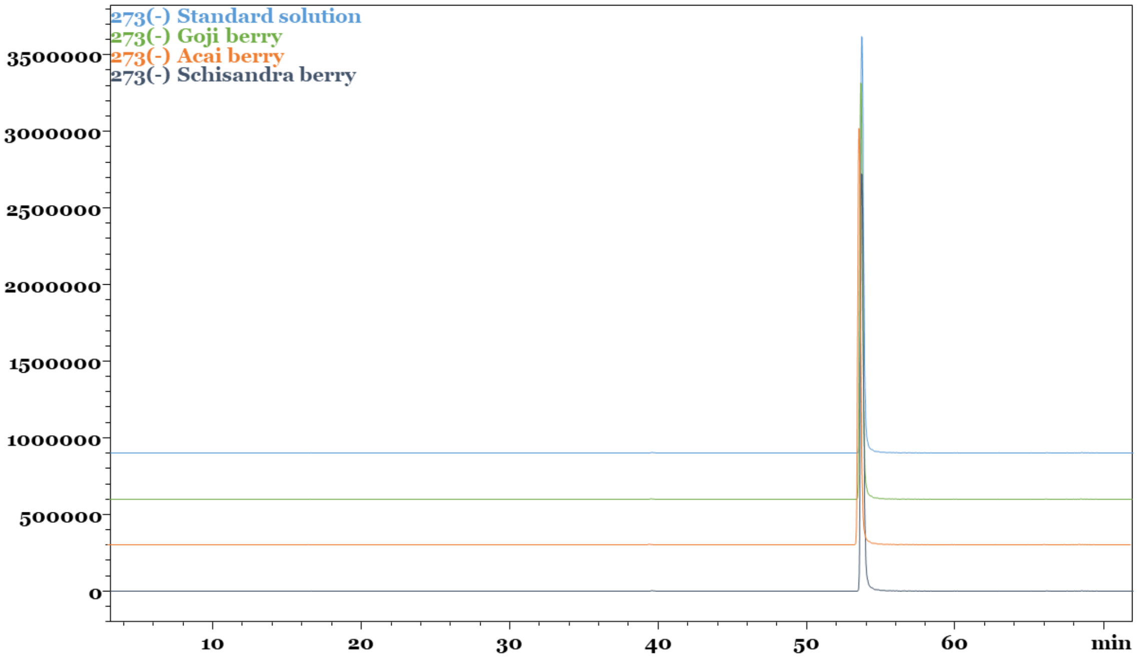

Figure S6. LC-MS (SIM) chromatogram recorded in negative mode, of an aqueous extracts of acai, goji and schisandra berries compared with standard solution of chlorogenic acid.

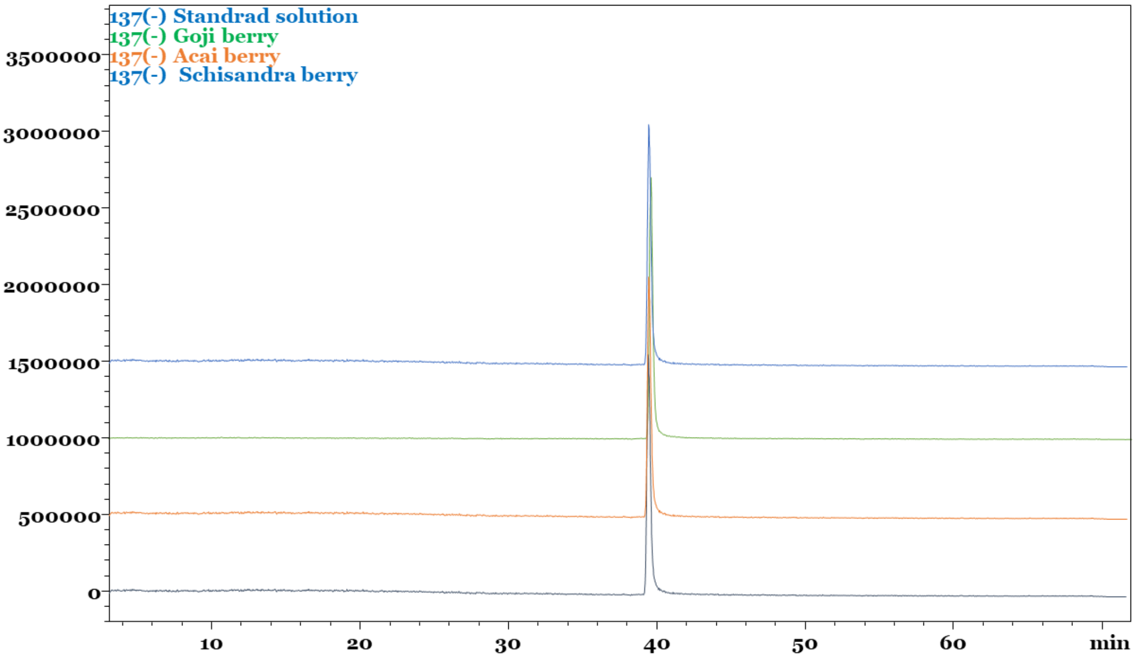

Figure S7. LC-MS (SIM) chromatogram recorded in negative mode, of an aqueous extracts of acai, goji and schisandra berries compared with standard solution of salicylic acid.

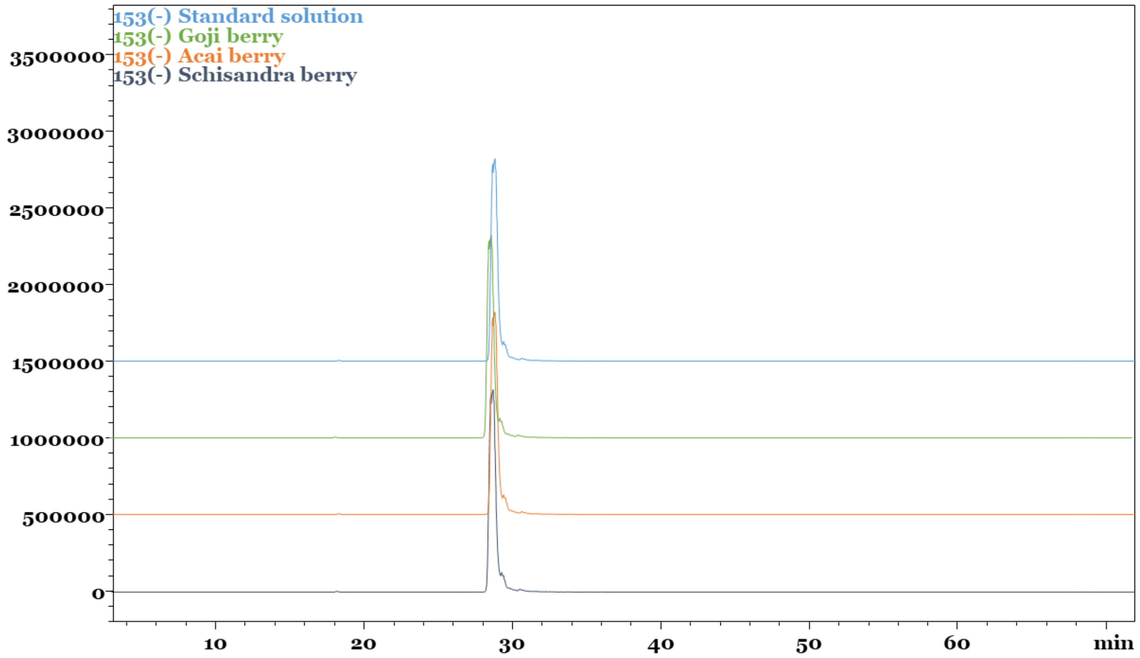

Figure S8. LC-MS (SIM) chromatogram recorded in negative mode, of an aqueous extracts of acai, goji and schisandra berries compared with standard solution of 2,4-diOH-benzoic acid.

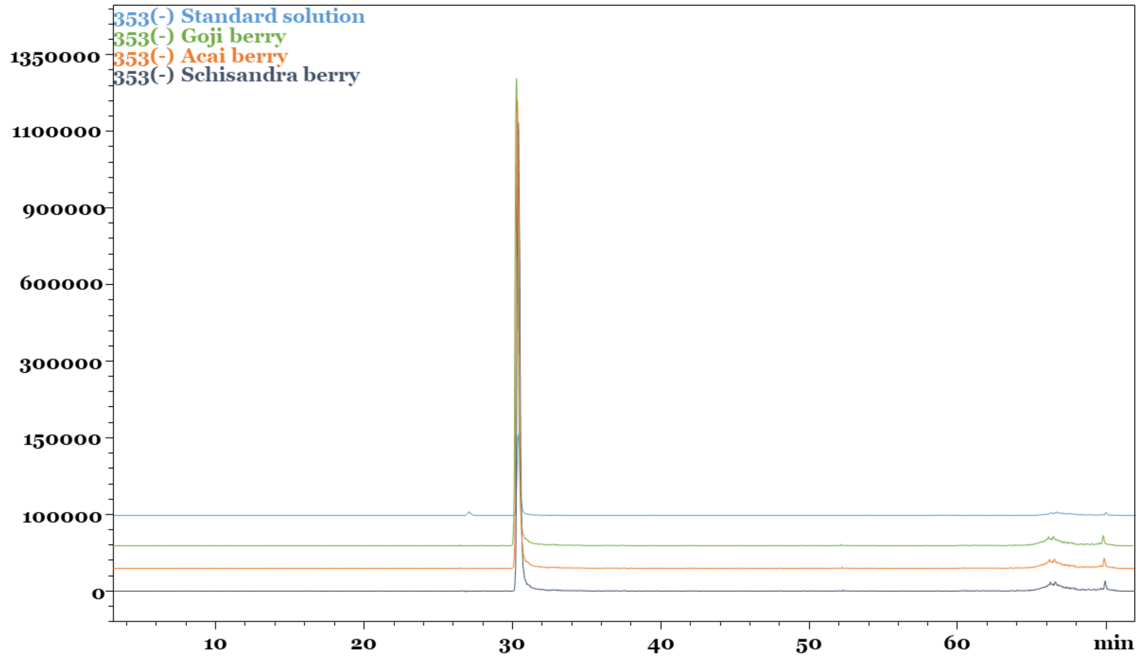

Figure S9. LC-MS (SIM) chromatogram recorded in negative mode, of an aqueous extracts of acai, goji and schisandra berries compared with standard solution of rutin.
